# Self-reported history of sexually transmissible infections (STIs) and STI-related utilization of the German health care system by men who have sex with men: data from a large convenience sample

**DOI:** 10.1186/1471-2334-11-132

**Published:** 2011-05-18

**Authors:** Axel J Schmidt, Ulrich Marcus

**Affiliations:** 1 Robert Koch Institute, Department for Infectious Diseases Epidemiology, Berlin, Germany

**Keywords:** Sexual Behaviour [MeSH], Delivery of Health Care [MeSH], Health Surveys [MeSH], Sexually Transmitted Diseases [MeSH], Men who have sex with men (MSM) [non-MeSH]

## Abstract

**Background:**

In Germany, testing and treatment of sexually transmissible infections (STIs) services are not provided by one medical discipline, but rather dispersed among many different providers. Common STIs like gonorrhoea or Chlamydia infection are not routinely reported. Although men who have sex with men (MSM) are particularly vulnerable to STIs, respective health care utilization among MSM is largely unknown.

**Methods:**

A sexual behaviour survey among MSM was conducted in 2006. Questions on self-reported sexual behaviour, STI-related health care consultation and barriers to access, coverage of vaccination against hepatitis, screening for asymptomatic STIs, self-reported history of STIs, and partner notification were analysed. Analysis was stratified by HIV-serostatus (3,511 HIV-negative/unknown versus 874 positive).

**Results:**

General Practitioners, particularly gay doctors, were preferred for STI-related health care. Low threshold testing in sex-associated venues was acceptable for most respondents. Shame and fear of homophobic reactions were the main barriers for STI-testing. More than half of the respondents reported vaccination against hepatitis A/B. HIV-positive MSM reported screening offers for STIs three to seven times more often than HIV-negative or untested MSM. Unlike testing for syphilis or hepatitis C, screening for asymptomatic pharyngeal and rectal infections was rarely offered. STIs in the previous twelve months were reported by 7.1% of HIV-negative/untested, and 34.7% of HIV-positive respondents.

**Conclusions:**

Self-reported histories of STIs in MSM convenience samples differ significantly by HIV-serostatus. Higher rates of STIs among HIV-positive MSM may partly be explained by more testing. Communication between health care providers and their clients about sexuality, sexual practices, and sexual risks should be improved. A comprehensive STI screening policy for MSM is needed.

## Background

In Germany, it is difficult to measure the burden of sexually transmissible infections (STIs), particularly among hidden or marginalised sub-populations like men who have sex with men (MSM).

First, STI diagnosis and therapy services are not concentrated in Genito-Urinary Medicine (GUM) or STI clinics (like e.g. in the UK), but dispersed among different medical disciplines: general practitioners (GPs), dermatologists/venerologists, urologists, and private or hospital-attached HIV outpatient clinics with a large clientele of MSM. Last but not least, German Public Health Offices (PHOs) historically offer anonymous counselling and testing for some STIs, including HIV.

Second, the German infectious diseases surveillance system was altered in 2001, and since then mandates reporting of only a few STIs: HIV, syphilis and acute hepatitis B virus infection. An additional sentinel system for STI surveillance was established in 2002; but results are highly biased [1].

Since 2001, increasing numbers of newly diagnosed syphilis and HIV infections among MSM have been observed [2,3]. This prompted us to plan a national survey on knowledge, attitudes, and behaviour as to sexually transmissible infections (KABaSTI) among MSM in Germany, piloting the establishment of a system of second generation HIV/STI surveillance in MSM. Due to reports suggesting that MSM with diagnosed HIV infection are particularly affected by other STIs [4] we decided to oversample HIV positive MSM by choosing health care facilities and a bareback website as study recruitment sites in addition to general chat and dating websites for MSM. With this survey we aimed to monitor STI-related knowledge, sexual behaviour, STI risk due to sexual network characteristics, STI-related health care consultation and barriers to access, coverage of vaccination against hepatitis, screening for asymptomatic STIs, self-reported recent and lifetime history of STIs, and partner notification.

## Methods

The survey was conducted in 2006, using an anonymous, self-administered, 66-item questionnaire. Details have been described elsewhere [5]. In summary, seven German language chat and dating websites for MSM provided links to an online questionnaire, including a website particularly for gay men who prefer anal sex without condoms ('bareback website'). In addition, participants were enrolled offline in 76 private medical practices or out-patient clinics who - based on syphilis notifications - deal with a high proportion of MSM among their patients. Participating medical facilities received print questionnaires for distribution to all recognizable male homosexual or bisexual clients. Participants were asked to return the questionnaire anonymously and in a prepaid envelope. No incentives were provided for completing the questionnaire.

For this report, we analysed questions on (1) sexual behaviour and diagnosed STIs or HIV among sexual partners, (2) STI-related health care consultation and barriers to access (3), coverage of hepatitis A/B vaccination, (4) diagnostic coverage of asymptomatic STIs (screening), (5) history of various STIs during lifetime and in the previous twelve months (referred to as 'recent' diagnoses), and (6) partner notification (for an English translation of the questions see Additional file 1). Analysis of online respondents was restricted to completed datasets. Analyses were stratified by self-reported HIV status, and odds ratios were calculated to compare respondents who were diagnosed HIV-positive with those who were not. To evaluate the impact of HIV serostatus on STI-screening, we conducted logistic multivariate regression analysis using IBM^® ^SPSS^® ^Statistics 18. Diagnosed STIs were additionally stratified by recruitment site.

## Results

Among the total of 4,385 respondents eligible for analysis, 3,511 reported a negative last HIV test result or no previous HIV test, and 874 reported a positive HIV test result. HIV-negative and untested men were predominantly recruited on general MSM chat and dating websites (n = 3,050), while large proportions of HIV-positive respondents were recruited on a 'bareback' website (n = 240), and in medical facilities (n = 417). Other socio-demographic data on the two subsamples are reported in table 1.

**Table 1:** Demographic characteristics and sexual behaviour in two sub-samples of MSM in Germany: n (%)

	Last HIV test negative or not tested for HIV n = 3,511	HIV-positive n = 874	OR (95%-CI)/p
***Recruitment and Demographics***			

Recruitment			<0.001 (χ^2^)
Chat- and Dating Websites	3,050 (86.9)	217 (24.8)	
'Bareback' Website	207 (5.9)	240 (27.5)	
Medical Facilities	254 (7.2)	417 (47.7)	

Median age (range)	32 (16; 76)	40 (20; 70)	>0.001 (t)

City size (population >500,000)	1,218 (34.7)	467 (53.4)	2.16 (1.86-2.51)

Education (ISCED 4 or higher)	1,836 (52.3)	414 (47.4)	0.82 (0.71-0.95)

***Sexual behaviour in the previous 12 months***			
More than ten sexual partners	974 (27.7)	466 (53.3)	2.98 (2.56-3.64)
Sex with non-steady partners was...			
frequently anally insertive	820 (23.4)	337 (38.6)	2.06 (1.76-2.41)
frequently anally receptive	792 (22.6)	382 (43.7)	2.67 (2.82-3.11)
Frequent HIV risk-taking*			
with male partners in general	222 (6.3)	233 (26.7)	5.39 (4.40-6.59)

with anonymous partners	135 (3.8)	208 (23.8)	7.81 (6.19-9.85)

***Sexual Networks: Reporting sexual partners with...***			
HIV	209 (6.0)	425 (48.6)	14.96 (12.33-18.13)
recent STIs**	178 (5.1)	192 (22.0)	5.27 (4.23-6.57)

*Frequent risk-taking: Five or more episodes of unprotected anal intercourse with a non-steady sexual partner of unknown or discordant HIV serostatus in the previous twelve months; **Current sexual partners with recent STIs: diagnosed with syphilis, gonorrhoea, or Chlamydia infection in the previous twelve months.

No differences were found between drop outs (i.e. respondents with incomplete data; n = 2,265) and the HIV-negative/untested group of respondents with respect to age, city-size, education, being recruited on a 'bareback' website, or frequency of anal intercourse with non-steady partners; drop outs had slightly fewer sexual partners (24.6% vs. 27.7% with more than 10 partners).

Within the HIV-negative/untested group, respondents who were never tested for HIV (n = 1,144) were younger, living in smaller cities, had lower educational degrees, and reported fewer sexual partners, less unprotected sex, and fewer STIs. Despite this heterogeneity they were grouped together with the HIV-negative respondents, as from the health care point of view, MSM who had a negative HIV test in the past are not regarded as different from MSM who did not, while screening and recommendations regarding some diagnostic procedures differ for MSM who are HIV-positive.

### (1) Sexual behaviour and diagnosed STIs or HIV among sexual partners

The proportion of respondents with more than ten sexual partners in the previous twelve months (table 1) was higher among HIV-positive MSM as compared to HIV-negative or untested MSM; the same holds true for frequently engaging in anal sex (both receptive and insertive), and for engaging in anal sex without a condom. More than a quarter of the HIV-positive and 6.3% of the HIV-negative/untested MSM reported five or more episodes of unprotected anal intercourse (UAI) with a partner of unknown (or discordant) HIV serostatus during the last twelve months ('frequent risk-taking'). Most of these reported risky sexual encounters occurred in settings where sexual partners were anonymous.

As the risk of acquiring an STI is the result of both an individual's sexual behaviour and the prevalence of STIs within his sexual networks, we asked for sexual partners who were known to be HIV positive or to have been diagnosed with syphilis, gonorrhoea, or Chlamydia infection in the previous 12 months. HIV-positive respondents reported not only a substantially higher proportion of sexual partners with HIV, but also a higher proportion of sexual partners with recent STIs than HIV-negative/untested respondents.

### (2) STI-related health care consultation and barriers to access

If suspecting an STI, about half of the respondents in both serostatus groups said they would visit a General Practitioner (table 2). Compared to HIV-positive MSM, the proportion of HIV-negative/untested MSM who chose the possibility of anonymous STI consultation at a Public Health Office (PHO) was twice as high. Almost 80% of HIV-negative/untested respondents would consider low threshold STI-testing in bars, discos, or saunas, if offered, but only 60.3% of HIV-positive respondents.

**Table 2:** STI-related health care, coverage of hepatitis A/B vaccination, screening for STIs, diagnosed STIs, and partner notification in two sub-samples of MSM in Germany: n (%); results for lifetime and the previous 12 months

	Last HIV test negative or not tested for HIV n = 3,511		HIV-positive n = 874		OR (95%-CI)/p
***STI-related health care***					
Preferred STI care provider					<0.001 (χ^2^)
General Practitioner	1,637 (46.6)		448 (51.3)		
Dermatologist	590 (16.8)		243 (27.8)		
Urologist	808 (23.0)		118 (13.5)		
Public Health office	475 (13.5)		55 (6.3)		
Low threshold testing*	2,806 (79.9)		527 (60.3)		0.38 (0.33-0.45)
Would prefer a gay physician	2,529 (72.0)		614 (70.3)		0.92 (0.78-1.08)
Communication barriers**	1,589 (45.3)		180(20.6)		0.31 (0.26-0.37)

	*Last 12 m.*	*Lifetime*	*Last 12 m.*	*Lifetime*	

Medical consultation for STIs	757 (21.6)		433 (49.5)		3.57 (3.06-4.17)
		1,327 (37.8)		597 (68.3)	3.55 (3.03-4.15)
No medical consultation***	190 (5.4)		28 (3.2)		0.58 (0.39-0.87)
Hepatitis A vaccination		1,932 (55.0)		566 (64.8)	1.50 (1.23-1.75)
Hepatitis B vaccination		2,045 (58.8)		577 (66.0)	1.39 (1.19-1.63)

***Self-reported testing/screening***^***§***^					

	*Last 12 m.*	*Lifetime*	*Last 12 m.*	*Lifetime*	

HIV	888 (25.3)^§§^	2,367 (67.4)	183 (20.9)^§§^	n.a.	n.a.
HCV	787 (22.4)		437 (50.0)		3.46 (2.97-4.04)
		1,223 (34.8)		577 (66.0)	3.64 (3.11-4.25)
Chlamydia	264 (7.5)		220 (25.2)		4.14 (3.40-5.04)
		415 (11.8)		300 (34.4)	3.90 (3.28-4.64)
Syphilis	556 (15.8)		472 (54.0)		6.24 (5.31-7.33)
		799 (22.8)		584 (66.8)	6.84 (5.82-8.03)
Gonorrhoea (rectal)	240 (6.8)		177 (20.3)		3.46 (2.90-4.27)
		352 (10.0)		248 (28.4)	3.56 (2.96-4.27)
Gonorrhoea (pharyngeal)	232 (6.6)		159 (18.2)		3.14 (2.53-3.91)
		330 (9.4)		223 (25.5)	3.30 (2.73-3.99)

Any of the above (not HIV)	992 (28.3)		572 (65.4)		4.81 (4.11-5.63)
		1,522 (43.3)		713 (81.6)	5.79 (4.82-6.95)

***Self-reported diagnoses***					

	*Last 12 m.*	*Lifetime*	*Last 12 m.*	*Lifetime*	

HCV	13 (0.4)		38 (4.3)		12.23 (6.49-23.06)
		29 (0.8)		77 (8.8)	11.60 (7.52-17.90)
Chlamydia	66 (1.9)		104 (11.9)		7.05 (5.13-9.69)
		199 (5.7)		233 (26.7)	6.05 (4.92-7.44)
Syphilis	84 (2.4)		138 (15.8)		7.65 (5.77-10.15)
		232 (6.6)		366 (41.9)	10.18 (8.43-12.30)
Gonorrhoea (urethral)	105 (3.0)		117 (13.4)		5.01 (3.81-6.60)
		459 (13.1)		357 (40.8)	4.59 (3.89-5.43)
Gonorrhoea (rectal)	28 (0.8)		39 (4.5)		5.81 (3.56-9.50)
		66 (1.9(		127 (14.5)	8.87 (6.52-12.07)
Gonorrhoea (pharyngeal)	12 (0.3)		17 (1.9)		5.78 (2.75-12.15)
		37 (1.1)		39 (4.5)	4.39 (2.78-6.92)

Any of the above (not HIV)	249 (7.1)		303 (34.7)		6.95 (5.75-8.40)
		745 (21.2)		616 (70.5)	8.87 (7.51-10.47)
Of those: Informed partner(s)	125 (50.2)		152 (50.2)		1.0 (0.71-1.39)

*Low threshold testing: No general objection to testing campaigns in bars, discos, or saunas; **Communication barriers: Respondents who said the most important reason for not consulting a health care provider although suspecting to have acquired an STI was 'shame', or a feeling that talking about sex in general, or about sex with men, was not possible with their health care provider(s); *** *No medical consultation*: Proportion of respondents who said they did not consult a health care provider despite having symptoms or knowing that a sexual partner had an STI; ^§ ^*Self-reported testing/screening*: Latest HIV antibody test; tested for STIs in the absence of specific symptoms? ^§§ ^Latest HIV test in 2005 or 2006 (i.e. within the previous 18 months); assuming that HIV-testing is equally spread over the year, this value was adjusted to 12 months

The main barrier for not seeking medical treatment was 'shame' (44.0%). Other frequently mentioned barriers were men's inability to talk to their physician about having sex with men (37.0%), or discomfort with talking about sex in general (23.0%). 'Shame' and the perceived inability to talk about sex with men were strongly correlated with city size - and most pronounced among respondents residing in rural areas or small towns. Taken together, communication issues as barriers for health care consultation were reported by 45.3% of HIV-negative/untested and 20.6% of HIV-positive respondents (table 2). Among online respondents, previous experience with STIs (and thus with medical personnel) lowered both fear and shame. However, three in four respondents expressed a preference for consulting an STI doctor who is gay - particularly MSM recruited in medical facilities, among whom perceived communication barriers were lowest (17.2%).

Financial constraints were least frequently reported as a barrier for seeing a physician (15.2%). Reporting financial barriers increased with declining incomes (retired: 20%; workers: 22%; unemployed: 33%).

### (3) Coverage of Hepatitis A/B vaccination

A majority of respondents in both serostatus groups reported being vaccinated against hepatitis A and B (table 2). Hepatitis vaccination status was negatively correlated with age: In Germany, vaccination against hepatitis B has been recommended for children and adolescents since 1995, and costs are covered by statutory health insurances. Among respondents 44 years of age or older 49% reported being vaccinated against hepatitis B (hepatitis A: 48%), while among those younger than 20 years of age the respective proportion was 79% (hepatitis A: 73%).

### (4) Diagnostic coverage of asymptomatic STIs (screening)

Of MSM reporting no positive HIV diagnosis, 67.4% had ever tested for HIV and 25.3% were tested recently, in the previous 12 months (table 2). Among non-HIV-positive men, one in four respondents from general MSM websites underwent HIV-testing in the previous twelve months, while among men recruited on a 'bareback website' or in medical facilities, it was one in two. Lifetime and recent history of STI-screening differed substantially between HIV-positive and HIV-negative/untested MSM. Moreover, being screened for STIs was more likely to be offered if possible to be done by serological testing. Screening for STIs requiring swab-testing or microbiological culturing for diagnosis, such as rectal or pharyngeal infections with Chlamydia or Gonococci, was only half as likely as for STIs requiring serological testing (table 2).

HIV serostatus, not sexual behaviour, was the key determinant of reported screening. Among HIV-positive respondents reporting sexual contacts with only one man in the last twelve months, 65.6% were screened in the past year for STIs other than HIV. In contrast, of respondents without HIV diagnosis, but with more than fifty sexual partners in the last twelve months, the proportion screened for other STIs was only 28.2%. Even if the latter group was restricted to men who reported recent testing for HIV, and thus a history of HIV-related risk-taking and contact with the health care system, less than half of them were simultaneously tested for syphilis (46.0%), hepatitis C (45.1%), Chlamydia infection (25.3%), pharyngeal gonorrhoea (18.7%), or rectal gonorrhoea (17.6%).

In multivariate regression analysis, HIV-serostatus was the strongest predictor for STI-screening in the previous 12 months (OR = 3.8; 95% confidence interval: 3.2-4.5) followed by the number of sexual partners (reference: 0-1 sexual partners in the last 12 months): 2-10 partners (1.5; 1.3-1.8), 11-20 partners (2.1; 1.6-2.6), and more than 20 partners (2.6; 2.1-3.2). Other independently associated factors included being 25 years of age or older (1.3; 1.1-1.6), living in a city with more than 500,000 inhabitants (1.2; 1.1-1.4), or frequently engaging in receptive anal intercourse with non-steady partners (1.2; 1.0-1.4). On the other hand, frequently engaging in insertive anal intercourse with non-steady partners, reporting unprotected anal intercourse with non-steady or anonymous partners of unknown or discordant HIV-status, a general positive attitude towards condom use for STI prevention, or education, were not associated with STI-screening.

### (5) Self-reported STIs: lifetime and previous 12 months

Recently diagnosed HIV infections were most frequent among respondents from the 'bareback website' (13.7%); among MSM from other websites, the respective value was a magnitude lower (1.7%).

The most frequently reported STI was urethral gonorrhoea, followed by syphilis. Rectal and pharyngeal infections with Gonococci were reported as 3-4 times less frequent. All recently diagnosed STIs - particularly hepatitis C and syphilis - were substantially more frequent among HIV-positive respondents, as were lifetime diagnoses of STIs (table 2). Higher proportions of respondents recruited on a 'bareback' website reported recently diagnosed STIs, even if compared with respondents recruited in medical facilities who had a higher prevalence of diagnosed HIV infection (table 3).

**Table 3:** Diagnosed STIs, stratified by recruitment method: n (%); results for lifetime and the previous 12 months

	Chat- & Dating Sites n = 3,267		'Bareback' website n = 447		Medical Facilities n = 671	
	***Last 12 m.***	***Lifetime***	***Last 12 m.***	***Lifetime***	***Last 12 m.***	***Lifetime***

HIV	55 (1.7)^§^	217 (6.6)	61 (13,7)^§^	240 (53.7)	68 (10,1)^§^	417 (62.1)
HCV	16 (0.5)	33 (1.0)	16 (3.6)	27 (6.0)	19 (2.8)	46 (6.9)
Chlamydia	69 (2.1)	191 (5.8)	46 (10.3)	106 (23.7)	55 (8.2)	135 (20.1)
Syphilis	81 (2.5)	238 (7.3)	77 (17.2)	169 (37.8)	64 (9.5)	191 (28.5)
Gonorrhoea (urethral)	101 (3.1)	425 (13.0)	74 (16.6)	180 (40.3)	47 (7.0)	211 (31.4)
Gonorrhoea (rectal)	26 (0.8)	76 (2.3)	19 (4.3)	49 (11.0)	22 (3.3)	68 (10.1)
Gonorrhoea (pharyngeal)	14 (0.4)	36 (1.1)	5 (1.1)	20 (4.5)	10 (1.5)	20 (3.0

Any of the above (not HIV)	233 (7.1)	688 (21.1)	167 (37.4)	293 (65.5)	152 (22.7)	380 (56.6)
Of those: Informed partner(s)	98 (42.1)		68 (40.7)		111 (73.0)	

^§ ^Latest HIV test in 2005 or 2006 (i.e. within the previous 18 months); assuming that HIV-testing is equally spread over the year, this value was adjusted to 12 months

### (6) Partner notification (PN)

Of those who reported a recent diagnosis of one of the abovementioned STIs (not HIV), 50.2% said they had informed their sexual partner(s). The most important reason for *non*-notification was not 'shame' (15.6%), or carelessness (13.2%), but the anonymity of many sexual partners (82.0%). Informing others about diagnosed STIs was highly associated with reporting that they had been informed by sexual partners about STI diagnoses (OR = 3.8; 95%-CI: 3.0-4.9). No differences were found between the Eastern and Western part of Germany. Respondents recruited in medical facilities were much more likely to report PN than respondents recruited online (table 3).

## Discussion

We found high rates of diagnosed STIs - during lifetime and the previous twelve months - among MSM recruited on chat and dating websites and in medical facilities, although testing rates for STIs in our sample tend to be lower than in published samples from other countries [6,7].

However, since the sample is a convenience sample, these rates cannot be expected to be representative of the German gay or MSM population. A self-selection bias favouring men with increased risk for HIV and STIs has to be expected.

**Figure 1:**
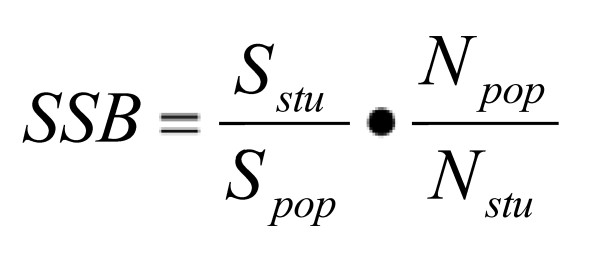
**Assessment of self-selection bias**. SSB self-selection bias; S_stu_, S_pop_: self-reported Syphilis diagnoses in the last 12 months in the study (stu)/Syphilis cases attributed to MSM in the primary surveillance data (pop); N_stu_, N_pop_: Number of MSM in the study (stu)/in the German population (pop), the latter based on extrapolations from sexual behaviour surveys of the general population.

Based on estimates of the total size of the identifiable MSM population in Germany [8], data from German STI-sentinels, and biological surveillance data, expected proportions of recent syphilis diagnoses among MSM with/without HIV infection can be calculated. If these proportions are compared to the respective proportions of reported recent syphilis among MSM with/without HIV infection, the level of self-selection bias can be assessed, using a calculation to measure the self-selection factor (figure 1).

When entering German data into this formula, self-selection bias results in factor 3-5 for HIV-positive respondents and in factor 10 for HIV-negative respondents. However, even taking this considerable self-selection bias into account, the burden of STIs in the MSM population is still significantly higher than in the heterosexual population.

In addition, other research has shown that among MSM, non-genital manifestations of gonorrhoea and Chlamydia infection are at least as frequent as genital manifestations [9], and that the majority of bacterial STIs are present without noticeable symptoms [9-11]. Since systematic screening for pharyngeal and rectal infections was quite rare in our sample, rectal/pharyngeal gonorrhoea and rectal/urethral Chlamydia infections are almost certainly underdiagnosed.

The comparatively minor role of reported Chlamydia infections can further be attributed to considerable knowledge gaps regarding Chlamydia among German MSM. Moreover, a widespread clinical practice of syndromic treatment for suspected Chlamydia infections might preclude a sound diagnostic approach.

The differences between STI diagnosis rates among HIV-positive and HIV-negative/untested respondents are substantial. However, since many of the STIs we looked at in this analysis may be present without causing characteristic symptoms, the large differences between reported STI screening rates likely will have an impact on the frequencies of diagnoses.

The finding that HIV-infected MSM were more likely to be evaluated for STIs than their non-infected counterparts may suggest one of three things: 1) given their risk profile, they may be more likely to present with symptomatic STIs; 2) they may feel more comfortable with their HIV care provider than others feel with their providers and thus more willing to share concerns about risk behaviours and STIs; or 3) their HIV care providers may be more pro-active in assessing ongoing risk behaviours and suggesting STI testing among their clientele.

Higher STI screening rates among HIV-positive patients in Germany are likely due to more intense contact with health care providers and fewer reimbursement problems if screening tests are prescribed in the context of health care provision for HIV infection. However, even for usually symptomatic STIs, such as urethral gonorrhoea, for which numbers of diagnoses are less biased by screening activities, a several-fold higher disease burden is reported by HIV-positive MSM compared to HIV-negative/untested men. Reasons for these differences may be related to higher partner numbers and less consistent condom use among HIV positive men. The role of HIV-serosorting has been analysed and discussed elsewhere [12].

Other biases in our results are related to sample characteristics: MSM recruited in medical facilities by definition already have access to STI-related care (60% among those medical facilities were specialised in HIV treatment). Results from this sub-sample therefore are likely to overestimate the burden of diagnosed STIs, particularly among HIV-negative MSM, and to underestimate specific barriers towards STI-related health care.

We are not able to define response rates for participants recruited on different websites, but we reach very different populations on general MSM and 'bareback' websites. Due to the higher numbers of sexual partners and the higher frequency of unprotected anal intercourse among MSM recruited on a 'bareback website', this sub-sample may represent an important part of 'STI core groups' among MSM. Such core groups are necessary to maintain continuous circulation of bacterial STIs, which usually have a limited period of infectiousness and can easily be cured by antibiotic treatment (table 3).

Experiences with partner notification (PN) in Germany are mixed: In the Eastern part, until re-unification in 1990, partner tracing for patients with STIs was mandatory; the Western policy relied on physicians encouraging patients to inform their sexual partner(s). In this survey, PN was relatively frequent, with no differences between East and West. For MSM, anonymity of sexual partners seems to be the main obstacle against PN. Given today's impact of Internet-based search for sexual partners, the use of 'new' technologies like SMS or chat room messages, as being reported from e.g. San Francisco or Sydney, should be implemented and evaluated for PN among MSM in Germany [13-15].

Although it is unclear to what extent respondents can distinguish between hepatitis A and hepatitis B, it is reassuring that the broad implementation of hepatitis B vaccination in 1995 [16] has resulted in high proportions of vaccinated MSM in younger age-groups. This is particularly reassuring, as the efficacy of hepatitis B vaccination *after *HIV infection is reduced [17].

Low threshold STI-testing at sex-associated gay venues is an option for improving the uptake of STI-testing; however, MSM at high risk for STIs expressed some reservations, particularly HIV-positive men. Concerns might be related to fears of loss of privacy, restrictive measures, or further stigmatization [18]. The acceptance of such testing approaches needs to be evaluated separately for different settings.

Approximately 90% of the German population is covered by statutory health insurance [19]. Except for a quarterly consultation fee (since 2004), these patients do not have to deal with costs of medical procedures, and are not used to paying for diagnostic tests. German statutory health insurance reimbursement rules for laboratory STI-testing include incentives only for notifiable STIs. This might explain why STI-screening as shown in our results is mainly restricted to syphilis, HIV, and HCV - although sexually acquired hepatitis C is highly uncommon among HIV-negative MSM [20]. With growing awareness about increasing HCV transmission among HIV-positive MSM, some health care providers seem to include HCV screening into routine health checks.

Although recommended (e.g. by the German STD society), screening for asymptomatic bacterial STIs, including pharyngeal and rectal swabs for Gonococci, or nucleic acid testing of urine or rectal swabs for Chlamydia, is thus rarely offered. It has been described repeatedly that GPs or infectious disease specialists tend to neglect sexual health care needs of at-risk patients or sexual minorities [6,21], beyond financial aspects. The discrepancy between over-testing HIV-positive gay men - even if presently at low risk for STIs - and not offering syphilis serology to MSM with many sexual partners is striking.

## Conclusions

The implementation of a comprehensive STI-screening program for sexually active MSM is urgently needed, but hampered by communication barriers. Talking about sexuality in general, about homosexual contacts in particular, or about marginalised, delicate, or MSM-specific sexual practices (frequent anal/oral intercourse with non-steady partners, 'rimming', 'fisting', etc.) is still an obstacle to adequate testing-strategies - particularly in smaller towns, or in health care facilities where MSM fear to be confronted with homophobic reactions. Training GPs in taking sexual histories, assessing sexual risks, and providing non-judgmental sexual health care would be a key step to improving sexual health care for MSM and other patients at risk for STIs [22].

## Competing interests

The authors declare that they have no competing interests.

## Authors' contributions

UM planned and supervised the study, AJS coordinated the study and performed all statistical analyses. AJS and UM drafted the manuscript with equal contributions. Both authors read and approved the final manuscript.

## Pre-publication history

The pre-publication history for this paper can be accessed here:

http://www.biomedcentral.com/1471-2334/11/132/prepub
